# Supplementary figures and images for: Comprehensive analysis of early T cell responses to acute Zika Virus infection during the first epidemic in Bahia, Brazil
Source: PLoS One. 2024 May 9;19(5):e0302684. doi: 10.1371/journal.pone.0302684 (PMC11081376; doi:10.1371/journal.pone.0302684)

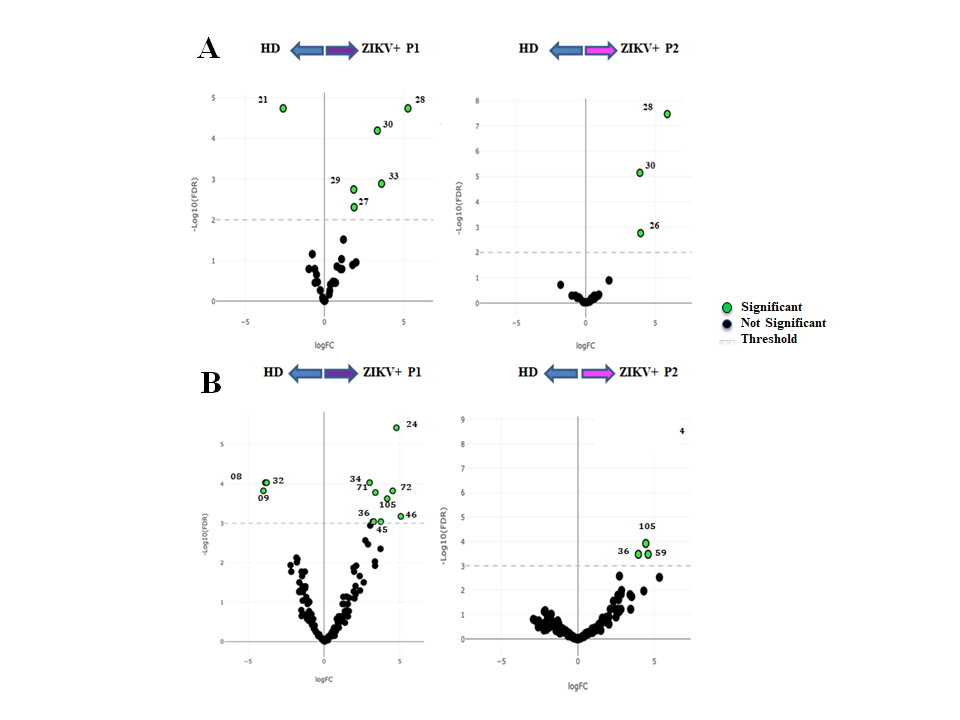

Supplement: S1 Fig — Analysis of clustering of CD4+ (A) and CD8+ (B) T cells by Volcano plot analysis from ZIKV-infected patients (ZIKV+) (N = 16) collected during the acute phase of the disease illness (P1) or during convalescent phase (P2) and from healthy donors (HD) (N = 10). Clusters that differ significantly between ZIKV+ and HD are indicated by green circles. (TIF) [file pone.0302684.s001.tif]

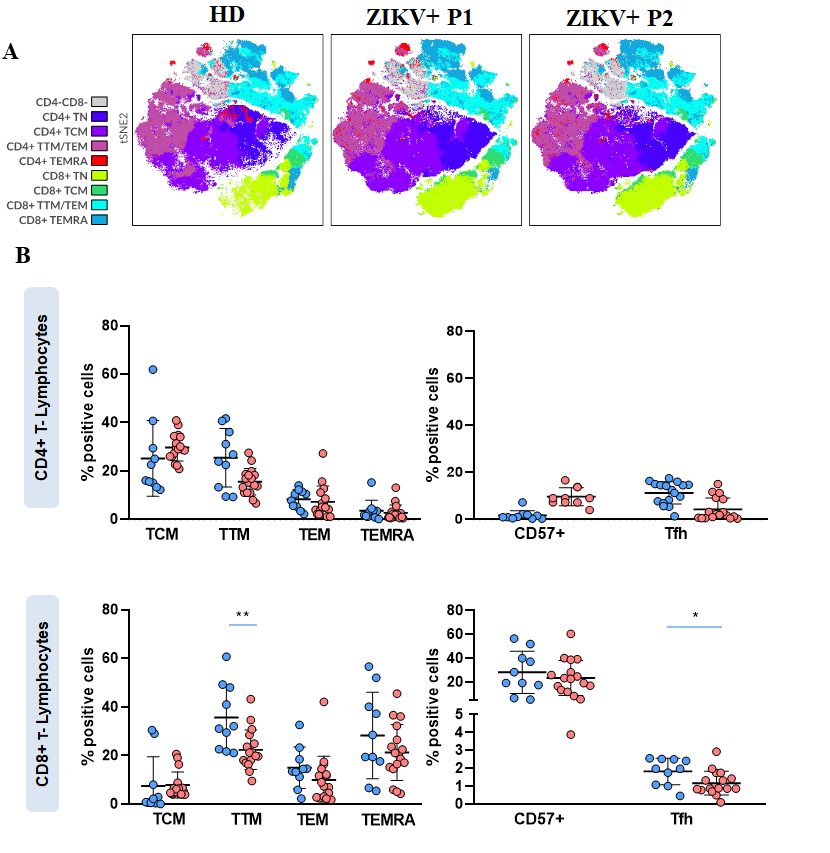

Supplement: S2 Fig — (A) Unsupervised tSNE analysis of naïve (TN), central memory (TCM), transitional memory (TTM), effector memory (TEM), and terminally differentiated effector memory (TEMRA) CD4+ and CD8+ T cells (B) Frequency of CD4+ and CD8+ memory subsets as well as CD57+ and T follicular helper cells (Tfh) in ZIKV-infected patients (ZIKV+; red circles) (N = 16) and healthy donors (HD; blue circles) (N = 10). Each circle in the graph represents an individual. The data are presented as the median and interquartile range. An unpaired Mann Whitney U test was conducted to compare HD and ZIKV+ patients, with significant differences denoted as follows: *p <0.05, **p <0.001. (TIF) [file pone.0302684.s002.tif]
