# Supplementary material for: Comprehensive analysis of early T cell responses to acute Zika Virus infection during the first epidemic in Bahia, Brazil
Source: PLoS One. 2024 May 9;19(5):e0302684. doi: 10.1371/journal.pone.0302684 (PMC11081376; doi:10.1371/journal.pone.0302684)
Supplement: S1 Table — (DOCX) [file pone.0302684.s003.docx]

**Supplemental Table 1: List of markers used in the study.**

| **Metal Tag** | **Target** | **Clone** | **Supplier** | **Parameters for tSNE** | **Parameters for FlowSOM (CD8 and CD4 Tcells)** |
| --- | --- | --- | --- | --- | --- |
| ^141^ Pr | CCR6 | GO34E3 | Fluidigm | Yes | Yes |
| ^142^ Nd | CD57 | HCD57 | Fluidigm | Yes | Yes |
| ^144^ Nd | CD38 | HIT2 | Fluidigm | Yes | Yes |
| ^145^ Nd | CD4 | RPA-T4 | Fluidigm | Yes | No |
| ^146^ Nd | CD8a | RPA-T8 | Fluidigm | Yes | No |
| ^162^ Dy | CD11c | Bu15 | Fluidigm | Yes | Yes |
| ^155^ Gd | CD27 | EPR8569 | Fluidigm | Yes | Yes |
| ^156^ Gd | CCR5 | NP-6G4 | Fluidigm | Yes | Yes |
| ^160^ Gd | CD14 | M5E2 | Fluidigm | No | No |
| ^163^ Dy | CD33 | WM53 | Fluidigm | No | No |
| ^164^ Dq | HLA-DR* | L243 | Fluidigm | Yes | Yes |
| ^166^ Er | NKG2C* | 134591 | R&D systems | No | Yes |
| ^167^ Er | KIR3DL1 | DX9 | Fluidigm | No | No |
| ^171^ Yb | CXCR5 | 51505 | Fluidigm | Yes | Yes |
| ^209^ Bi | CD11b | ICRF44 | Fluidigm | Yes | Yes |
| ^159^ Tb | CCR7 | GO43H7 | Fluidigm | Yes | Yes |
| ^143^ Nd | CD45Ra | HI100 | Fluidigm | Yes | Yes |
| ^149^ Sm | CD25 | EPR6452 | Fluidigm | Yes | Yes |
| ^154^ Sm | Tim3 | F38-2E2 | Fluidigm | Yes | Yes |
| ^115^ Ln | CX3CR1* | 2A9-1 | Fluidigm | No | No |
| ^148^ Nd | CD16 | 3G8 | Fluidigm | Yes | Yes |
| ^151^ Eu | CD123 | 6H6 | Fluidigm | No | No |
| ^165^ Ho | CD163 | GHI/61 | Fluidigm | No | No |
| ^174^ Yb | CD49b | P1E6-C5 | Fluidigm | No | No |
| ^175^ Lu | PD1 | EH12.2H7 | Fluidigm | Yes | Yes |
| ^150^ Nd | CD86 | IT2.2 | Fluidigm | No | No |
| ^161^ Dy | CD80 | 2D10.4 | Fluidigm | No | No |
| ^170^ Er | CTLA4 | 14D3 | Fluidigm | Yes | Yes |
| ^172^ Yb | Ki67 | Ki-67 | Fluidigm | Yes | Yes |
| ^168^ Er | IFN-γ | B27 | Fluidigm | Yes | Yes |
| ^152^ Sm | KIR2DL1* | REA248 | Miltenyi | No | Yes |
| ^173^ Yb | KIR2DL2/DL3 | DX-27 | Fluidigm | No | Yes |
| ^169^ Tm | NKG2A* | Z199 | Fluidigm | No | Yes |
| Qdot 605 | CD3 | UCHT1 | Fluidigm | Yes | No |
| Cisp 194 | CD19* | HIB19 | Fluidigm | No | No |
| Cisp 198 | CD66b* | 80H3 | Fluidigm | No | No |
| ^176^ Yb | CD56 | NCAM16.2 | Fluidigm | Yes | Yes |
| ^89^ Y | CD45 | HI30 | Fluidigm | No | No |
| ^153^ Eu | CCR2 | RMO52 | Fluidigm | No | Yes |
| ^147^ Sm | P-STAT5 | pY694 (47) | Fluidigm | Yes | Yes |

*: in-house mAb conjugated to metal isotopes
