# Supplementary material for: Comprehensive analysis of early T cell responses to acute Zika Virus infection during the first epidemic in Bahia, Brazil
Source: PLoS One. 2024 May 9;19(5):e0302684. doi: 10.1371/journal.pone.0302684 (PMC11081376; doi:10.1371/journal.pone.0302684)
Supplement: S2 Table — (DOCX) [file pone.0302684.s004.docx]

**Supplemental Table 2: HLA present in Brazil and Frequency in the population**

| **HLA A** | **% in the population** | **HLA B** | **% in the population** | **HLA C** | **% in the population** |
| --- | --- | --- | --- | --- | --- |
| A*0201 | 20.7 | B*3501 | 9 | Cw-0401 | 22.2 |
| A*0301 | 9.7 | B*4403 | 7.2 | Cw-0701 | 11.5 |
| A*2301 | 6.6 | B*5101 | 6.6 | Cw-1601 | 7.5 |
| A*0101 | 5.8 | B*0702 | 6.3 | Cw-0602 | 6.8 |
| A*3001 | 5.1 | B*5301 | 5.6 | Cw-0304 | 6.6 |
| A*1101 | 4.4 | B*1402 | 4.7 | Cw-0802 | 5.6 |
| A*2402 | 4.4 | B*4402 | 4.3 | Cw-0702 | 5 |
| A*6801 | 3.2 | B*5201 | 3.8 | Cw-0501 | 4.7 |
| A*3402 | 3.1 | B*1801 | 3.6 | Cw-1203 | 3.9 |
| A*3601 | 3.1 | B*0801 | 3 | Cw-0303 | 3.8 |
| A*6802 | 2.9 | B*5801 | 3 | Cw-0202 | 3.6 |
| A*7401 | 2.7 | B*4201 | 2.9 | Cw-1701 | 3.2 |
| A*3002 | 2.6 | B*1501 | 2.7 | Cw-0210 | 2.3 |
| A*2601 | 2.4 | B*1510 | 2.5 | Cw-1801 | 2.3 |
| A*3301 | 2 | B*4901 | 2.5 | Cw-1402 | 2 |
| A*0202 | 1.9 | B*1503 | 2 | Cw-1502 | 2 |
| A*2902 | 1.7 | B*4001 | 2 | Cw-0102 | 1.3 |
| A*3201 | 1.7 | B*8101 | 2 | Cw-1202 | 1.1 |
| A*3101 | 1.5 | B*5703 | 1.8 | Cw-1505 | 1.1 |
| A*0205 | 1.4 | B*4501 | 1.4 | Cw-1602 | 0.9 |
| A*6602 | 1.2 | B*3801 | 1.3 | Cw-0704 | 0.7 |
| A*2901 | 1 | B*4002 | 1.3 | Cw-0804 | 0.7 |
| A*8001 | 0.9 | B*5501 | 1.1 | Cw-0103 | 0.2 |
| A*3004 | 0.7 | B*5701 | 1.1 | Cw-0301 | 0.2 |
| A*0302 | 0.5 | B*5802 | 1.1 | Cw-0302 | 0.2 |
| A*2403 | 0.5 | B*1302 | 0.9 | Cw-0705 | 0.2 |
| A*2501 | 0.5 | B*1401 | 0.9 | Cw-0707 | 0.2 |
| A*3303 | 0.5 | B*1516 | 0.9 | Cw-0813 | 0.2 |
| A*0222 | 0.3 | B*2705 | 0.9 | Cw-1704 | 0.2 |
| A*0234 | 0.3 | B*3503 | 0.9 |  |  |
| A*6603 | 0.3 | B*3508 | 0.9 |  |  |
| A*7403 | 0.3 | B*4004 | 0.9 |  |  |
| A*6602 | 0.2 | B*5001 | 0.9 |  |  |
| A*0102 | 0.2 | B*0705 | 0.7 |  |  |
| A*0211 | 0.2 | B*1517 | 0.7 |  |  |
| A*6901 | 0.2 | B*3504 | 0.7 |  |  |
| A*7402 | 0.2 | B*3701 | 0.7 |  |  |
