# Supplementary material for: Comprehensive analysis of early T cell responses to acute Zika Virus infection during the first epidemic in Bahia, Brazil
Source: PLoS One. 2024 May 9;19(5):e0302684. doi: 10.1371/journal.pone.0302684 (PMC11081376; doi:10.1371/journal.pone.0302684)
Supplement: S3 Table — (DOCX) [file pone.0302684.s005.docx]

**Supplemental Table 3: Peptide sequence for Zika virus with predicted epitope and restriction element**

| **Protein** | **Amino Acid Sequence** | **Predicted Epitope** | **Restriction element** |
| --- | --- | --- | --- |
| **Capside** | VA**RVSPFGGLK**RLPA | RVSPFGGLK | **HLA-A*03:01; HLA-A*03:02; HLA-A*11:01** |
|  | G**LKRLPAGLLL**GHGP | KRLPAGLLL | **HLA-B*27:05** |
|  | HGPI**RMVLAILAFL**R | RMVLAILAF/MVLAILAFL | **HLA-C*07:01; HLA-C*07:05; HLA-B*15:01;HLA-B*15:03; HLA-C*03:02;HLA-C*07:02; HLA-C*07:07; HLA-C*16:01/HLA-A*68:02; HLA-A*69:01** |
|  | AI**LAFLRFTAI**KPSL | LAFLRFTAI | **HLA-B*08:01; HLA-B*52:01; HLA-C*01:02; HLA-C*07:04; HLA-C*18:01** |
|  | A**AMLRIINAR**KEKKR | AMLRIINAR | **HLA-A*74:01; HLA-A*74:02;HLA-A*74:03;HLA-A*31:01** |
| **Enveloppe** | SN**MAEVRSYCY**EASI | MAEVRSYCY | **HLA-A*01:01;HLA-A*01:02** |
|  | QPEN**LEYRIMLSV**HG | LEYRIMLSV | **HLA-B*13:02; HLA-B*45:01, HLA-B*49:01;HLA-B*50:01;HLA-B*52:01** |
|  | EPRT**GLDFSDLYY**LT | GLDFSDLYY | **HLA-A*01:01;HLA-A*36:01; HLA-A*80:01** |
|  | GLD**FSDLYYLTM**NNK | FSDLYYLTM | **HLA-A*01:01** |
|  | WLVH**KEWFHDIPL**PW | KEWFHDIPL | **HLA-B*40:01; HLA-B*40:02; HLA-B*40:04; HLA-B*37:01; HLA-B*50:01; HLA-B*13:02; HLA-B*38:01** |
|  | KEALV**EFKDAHAKR**Q | EFKDAHAKR | **HLA-A*33:01; HLA-A*33:03; HLA-A*32:01** |
|  | V**SYSLCTAAFTFTK**I | SYSLCTAAF/CTAAFTFTK | **HLA-A*24:03; HLA-A*24:02/HLA-A*03:02; HLA-A*11:01; HLA-A*34:02; HLA-A*68:01; HLA-A*74:01; HLA-A*74:02; HLA-A*74:03** |
|  | A**ETLHGTVTV**EVQYA | ETLHGTVTV | **HLA-A*68:02; HLA-A*69:01** |
|  | NSK**MMLELDPPF**GDS | MMLELDPPF | **HLA-C*07:02; HLA-C*07:01; HLA-C*07:05** |
|  | IVIGV**GEKKITHHW**H | GEKKITHHW | **HLA-B*44:02** |
|  | KR**MAVLGDTAW**DFGS | MAVLGDTAW | **HLA-B*53:01; HLA-B*58:01** |
|  | VLG**DTAWDFGSV**GGA | DTAWDFGSV | **HLA-A*68:02** |
| **NS1** | HPD**SPRRLAAAV**KQA | SPRRLAAAV | **HLA-B*07:02; HLA-B*07:05; HLA-B*42:01; HLA-B*55:01** |
|  | SVSR**MENIMWRSV**EG | MENIMWRSV | **HLA-B*45:01** |
|  | RGPQ**RLPVPVNEL**PH | RLPVPVNEL | **HLA-C*01:02** |
|  | FV**RAAKTNNSF**VVDG | RAAKTNNSF | **HLA-B*15:03;HLA-C*03:02; HLA-C*07:01; HLA-C*12:02; HLA-C*12:03; HLA-C*14:02** |
|  | CP**LEHRAWNSF**LVED | LEHRAWNSF | **HLA-B*18:01** |
|  | GFGV**FHTSVWLKV**RE | FHTSVWLKV | **HLA-B*38:01** |
|  | NDT**WRLKRAHLI**EMK | WRLKRAHLI | **HLA-B*27:05** |
|  | EMKT**CEWPKSHTL**WA | CEWPKSHTL | **HLA-B*37:01; HLA-B*13:02; HLA-B*38:01; HLA-B*40:02** |
|  | SDLI**IPKSLAGPL**SH | IPKSLAGPL | **HLA-B*07:02; HLA-B*07:05** |
|  | RSTT**ASGRVIEEW**CC | ASGRVIEEW | **HLA-B*57:01; HLA-B*58:01** |
|  | E**CTMPPLSFR**AKDGC | CTMPPLSFR | **HLA-A*33:03; HLA-A*33:01; HLA-A*68:01; HLA-A*74:01; HLA-A*74:02; HLA-A*74:03** |
|  | STS**MAVLVAMIL**GGF | MAVLVAMIL | **HLA-B*67:02** |
|  | AK**LAILMGATF**AEMN | LAILMGATF | **HLA-C*07:03** |
| **NS2A** | FKV**RPALLVSFI**FRA | RPALLVSFI | **HLA-B*42:04** |
|  | PALL**VSFIFRANW**TP | VSFIFRANW | **HLA-B*57:01; HLA-B*57:03; HLA-B*57:04; HLA-B*58:02; HLA-B*58:05** |
|  | GDL**MVLINGFAL**AWL | MVLINGFAL | **HLA-C*08:01; HLA-C*08:03** |
|  | VVP**RTDNITLAI**LAA | RTDNITLAI | **HLA-A*32:01; HLA-A*32:02; HLA-A*32:03** |
|  | **LPFVMALGLTAVR**LV | LPFVMALGL/MALGLTAVR/VMALGLTAV | **HLA-B*81:01; HLA-B*81:02; HLA-B*81:03; HLA-B*42:01; HLA-B*42:04; HLA-B*42:05; HLA-B*51:04; HLA-B*55:03; HLA-B*55:04; HLA-B*67:01; HLA-B*81:05; HLA-B*82:01;HLA-B*82:02/HLA-A*33:03/HLA-A*02:03** |
|  | SGK**RSWPPSEVL**TAV | RSWPPSEVL | **HLA-B*57:02; HLA-B*57:03; HLA-B*57:05** |
| **NS2B** | SWPP**SEVLTAVGL**IC | SEVLTAVGL | **HLA-B*40:01; HLA-B*40:04** |
|  | LL**IVSYVVSGK**SVDM | IVSYVVSGK | **HLA-A*34:02; HLA-A*34:03; HLA-A*34:04** |
|  | PM**REIILKVVL**MTIC | REIILKVVL | **HLA-B*40:01; HLA-B*40:02** |
|  | VVL**MTICGMNPI**AIP | MTICGMNPI | **HLA-A*25:02; HLA-A*25:03; HLA-A*25:04; HLA-A*68:02; HLA-A*69:01; HLA-C*15:02; HLA-C*15:03; HLA-A*34:01; HLA-A*34:05; HLA-C*08:01; HLA-C*08:03; HLA-C*12:02; HLA-C*15:05; HLA-C*17:01; HLA-C*17:02; HLA-C*17:02; HLA-C*17:03; HLA-C*17:04; HLA-C*17:05** |
|  | TIC**GMNPIAIPF**AAG | GMNPIAIPF | **HLA-B*15:03** |
|  | M**NPIAIPFAA**GAWYV | NPIAIPFAA | **HLA-B*54:01; HLA-B*54:02** |
|  | AIP**FAAGAWYVY**VKT | FAAGAWYVY/IPFAAGAWY | **HLA-B*18:04; HLA-B*35:01; HLA-B*35:05; HLA-B*46:01; HLA-B*46:02; HLA-B*46:04; HLA-B*46:05; HLA-B*53:03; HLA-B*56:03; HLA-C*02:03; HLA-C*02:04; HLA-C*02:05; HLA-C*03:02; HLA-C*05:04; HLA-C*06:03; HLA-C*12:02; HLA-C*12:03; HLA-C*12:04; HLA-C*12:05; HLA-C*15:04; HLA-C*16:01; HLA-C*16:02; HLA-C*16:04; HLA-A*29:03; HLA-A*29:04; HLA-B*15:02; HLA-B*35:03; HLA-B*46:03; HLA-B*53:01; HLA-B*53:02; HLA-B*53:05; HLA-B*83:01; HLA-C*01:04; HLA-C*07:01; HLA-C*07:03; HLA-C*15:02; HLA-C*15:03; HLA-A*29:01; HLA-A*29:02; HLA-C*06:02; HLA-C*07:02; HLA-C*14:05/HLA-B*35:01; HLA-B*56:03; HLA-B*83:01; HLA-B*35:05; HLA-B*53:03** |
|  | W**YVYVKTGKR**SGALW | YVYVKTGKR | **HLA-A*33:03** |
|  | KRSG**ALWDVPAPK**EV | ALWDVPAPK* | **HLA-A*03:02; HLA-A*03:07; HLA-A*74:01; HLA-A*74:02; HLA-A*74:03** |
|  | KG**ETTDGVYRV**MTRR | ETTDGVYRV* | **HLA-A*68:02; HLA-A*69:01** |
| **NS3** | TD**GVYRVMTRRLL**GS | GVYRVMTRR/YRVMTRRLL | **HLA-A*74:01; HLA-A*74:02; HLA-A*74:03; HLA-A*31:01/HLA-B*27:05** |
|  | T**RRLLGSTQV**GVGVM | RRLLGSTQV | **HLA-B*27:05** |
|  | HTM**WHVTKGSALR**SG | WHVTKGSAL/HVTKGSALR | **HLA-B*15:10; HLA-B*14:01; HLA-B*14:02/HLA-A*33:03; HLA-A*68:01; HLA-A*33:01; HLA-A*34:02** |
|  | PYWG**DVKQDLVSY**CG | DVKQDLVSY | **HLA-A*25:01; HLA-A*26:01** |
|  | VIKNGS**YVSAITQGR** | YVSAITQGR | **HLA-A*68:01** |
|  | FEPS**MLKKKQLTV**LD | MLKKKQLTV | **HLA-B*08:01** |
|  | EAI**KTRLRTVI**LAPT | KTRLRTVIL | **HLA-A*30:01** |
|  | MG**EAAAIFMTA**TPPG | EAAAIFMTA | **HLA-A*68:02** |
|  | AAIF**MTATPPGTR**DA | MTATPPGTR | **HLA-A*68:01; HLA-A*34:02; HLA-A*33:03** |
|  | RDA**FPDSNSPIM**DTE | FPDSNSPIM | **HLA-B*35:03; HLA-B*35:04; HLA-B*35:08** |
|  | EVPE**RAWSSGFDW**VT | RAWSSGFDW | **HLA-B*57:01; HLA-B*57:03; HLA-B*58:01, HLA-B*58:02, HLA-B*15:16** |
|  | HSG**KTVWFVPSVR**NG | KTVWFVPSV/TVWFVPSVR | **HLA-A*69:01/HLA-A*33:03; HLA-A*68:01; HLA-A*33:01; HLA-A*74:01; HLA-A*74:02; HLA-A*74:03; HLA-A*31:01; HLA-A*34:02** |
|  | SR**KTFETEFQK**TKHQ | KTFETEFQK | **HLA-A*74:01; HLA-A*74:02; HLA-A*74:03; HLA-A*03:02, HLA-A*11:01** |
|  | I**SEMGANFKA**DRVID | SEMGANFKA | **HLA-B*45:01** |
|  | AGP**MPVTHASAA**QRR | MPVTHASAA | **HLA-B*55:01; HLA-B*42:01** |
|  | WLEA**RMLLDNIYL**QD | RMLLDNIYL | **HLA-C*07:04** |
|  | EQR**KTFVELMKR**GDL | KTFVELMKR | **HLA-A*74:01; HLA-A*74:02; HLA-A*74:03** |
|  | GD**LPVWLAYQV**ASAG | LPVWLAYQV | **HLA-B*55:01; HLA-B*51:01** |
| **NS4A** | AQLP**ETLETIMLL**GL | ETLETIMLL | **HLA-A*69:01; HLA-A*68:02** |
|  | IG**KMGFGMVTL**GASA | KMGFGMVTL | **HLA-A*32:01** |
|  | G**TVSLGIFFV**LMRNK | TVSLGIFFV | **HLA-A*69:01; HLA-A*68:02** |
| **NS4B** | IDL**RPASAWAIY**AAL | RPASAWAIY | **HLA-B*35:01; HLA-C*14:02; HLA-B*35:08; HLA-C*06:02; HLA-C*07:07** |
|  | PA**SAWAIYAALTTF**I | SAWAIYAAL/AIYAALTTF | **HLA-C*01:02; HLA-C*01:03; HLA-C*07:01; HLA-C*07:02; HLA-C*07:04; HLA-C*08:04; HLA-C*08:13/ HLA-A*32:01; HLA-B*15:01** |
|  | TF**ITPAVQHAVTTSY** | ITPAVQHAV/VQHAVTTSY | **HLA-A*69:01/HLA-B*15:01; HLA-B*15:03** |
|  | VLF**GMGKGMPFY**AWD | GMGKGMPFY | **HLA-A*80:01** |
|  | MPF**YAWDFGVPL**LMI | YAWDFGVPL | **HLA-C*08:04; HLA-C*08:13; HLA-C*01:02; HLA-C*03:03; HLA-C*03:04; HLA-C*07:01; HLA-C*07:02; HLA-C*08:02; HLA-C*15:02; HLA-C*15:05; HLA-C*16:01; HLA-C*17:01; HLA-C*17:04; HLA-C*01:03; HLA-C*03:02; HLA-C*05:01; HLA-C*07:04; HLA-C*07:05; HLA-C*07:07** |
|  | AI**ILLVAHYMY**LIPG | ILLVAHYMY | **HLA-A*29:01; HLA-A*29:02** |
|  | YM**YLIPGLQAA**AARA | YLIPGLQAA | **HLA-A*02:05** |
|  | DID**TMTIDPQVEK**KM | MTIDPQVEK | **HLA-A*34:02; HLA-A*68:01; HLA-A*11:01** |
|  | IAV**AVSSAILSR**TAW | AVSSAILSR | **HLA-A*74:01; HLA-A*74:02; HLA-A*74:03** |
|  | W**GEAGALITA**ATSTL | GEAGALITA | **HLA-B*45:01** |
|  | GAL**ITAATSTLW**EGS | ITAATSTLW | **HLA-B*15:16; HLA-B*57:01; HLA-B*58:01; HLA-B*15:17; HLA-B*57:03; HLA-B*58:02** |
|  | RGS**YLAGASLIY**IVT | YLAGASLIY | **HLA-A*29:01; HLA-A*29:02; HLA-A*80:01; HLA-C*02:02; HLA-C*02:10** |
| **NS5** | NQ**MSALEFYSYK**KSG | MSALEFYSY/SALEFYSYK | **HLA-A*01:02; HLA-A*01:01; HLA-A*29:01; HLA-A*29:02; HLA-A*30:04; HLA-A*36:01; HLA-C*16:02; HLA-A*30:02; HLA-B*15:17; HLA-C*16:01/HLA-A*34:02; HLA-A*03:02; HLA-A*11:01; HLA-A*74:01; HLA-A*74:02; HLA-A*74:03** |
|  | L**VERGYLQPY**GKVID | VERGYLQPY | **HLA-B*18:01; HLA-B*44:02; HLA-B*50:01** |
|  | WNIV**RLKSGVDVF**HM | RLKSGVDVF | **HLA-B*15:01** |
|  | CDI**GESSSSPEV**EEA | GESSSSPEV | **HLA-B*45:01** |
|  | CPYTS**TMMETLERL**Q | TMMETLERL | **HLA-A*02:02; HLA-A*02:11; HLA-A*02:22; HLA-A*02:01; HLA-A*02:05** |
|  | RLQ**RRYGGGLVR**VPL | RRYGGGLVR | **HLA-B*27:05** |
|  | RNSTH**EMYWVSGAK**S | EMYWVSGAK | **HLA-A*34:02** |
|  | IR**SEHAETWFF**DENH | SEHAETWFF | **HLA-B*44:02; HLA-B*44:03; HLA-B*18:01** |
|  | HAET**WFFDENHPY**RT | WFFDENHPY | **HLA-A*29:01; HLA-A*29:02; HLA-A*30:04** |
|  | Y**RTWAYHGSY**EAPTQ | RTWAYHGSY | **HLA-A*30:02; HLA-A*30:04; HLA-B*15:17; HLA-A*29:01; HLA-A*29:02; HLA-A*32:01; HLA-A*80:01; HLA-B*15:16; HLA-A*01:02; HLA-A*74:01; HLA-A*74:02; HLA-A*74:03; HLA-C*01:03; HLA-C*03:02; HLA-C*06:02; HLA-C*07:01; HLA-C*07:07; HLA-C*14:02** |
|  | G**IAMTDTTPY**GQQRV | IAMTDTTPY | **HLA-B*35:01; HLA-C*02:02; HLA-C*02:10; HLA-C*03:02; HLA-C*12:02; HLA-C*12:03; HLA-C*16:01; HLA-C*16:02; HLA-B*35:08** |
|  | QV**MSMVSSWLW**KELG | MSMVSSWLW/SMVSSWLWK | **HLA-B*57:01; HLA-B*57:03; HLA-B*58:01; HLA-B*58:02; HLA-B*15:16; HLA-B*53:01; HLA-B*15:17/ HLA-A*03:01; HLA-A*03:02; HLA-A*11:01** |
|  | E**KEWKTAVEA**VNDPR | KEWKTAVEA | **HLA-B*45:01** |
|  | AK**GSRAIWYMW**LGAR | GSRAIWYMW | **HLA-B*57:01; HLA-B*58:02** |
|  | RAIW**YMWLGARFL**EF | YMWLGARFL | **HLA-C*07:04; HLA-C*07:01; HLA-C*07:02; HLA-C*07:05** |
|  | GARF**LEFEALGFL**NE | LEFEALGFL | **HLA-B*40:01; HLA-B*37:01; HLA-B*40:02; HLA-B*40:04** |
|  | IIK**YTYQNKVVKVL**R | YTYQNKVVK/YQNKVVKVL | **HLA-A*34:02/HLA-B*15:10** |
|  | QVVT**YALNTFTNL**VV | YALNTFTNL | **HLA-C*07:04; HLA-C*08:04; HLA-C*08:13; HLA-C*01:02; HLA-C*03:03; HLA-C*03:04; HLA-C*07:02; HLA-C*08:02; HLA-C*18:01; HLA-C*04:01; HLA-C*05:01; HLA-C*15:05; HLA-C*17:01;HLA-C*17:04** |
|  | EV**LEMQDLWLL**RRSE | LEMQDLWLL | **HLA-B*37:01; HLA-B*40:01; HLA-B*40:04; HLA-B*13:02; HLA-B*38:01; HLA-B*40:02; HLA-B*44:03; HLA-B*45:01; HLA-B*49:01** |
|  | T**QEWKPSTGW**DNWEE | QEWKPSTGW | **HLA-B*44:02; HLA-B*44:03** |
|  | A**KSYAQMWQLLYF**HR | KSYAQMWQL/SYAQMWQLL/YAQMWQLLY/AQMWQLLYF | **HLA-B*57:03; HLA-B*57:01; HLA-A*32:01; HLA-B*15:16; HLA-B*58:01/HLA-C*04:01/HLA-A*01:02; HLA-A*29:01; HLA-A*29:02; HLA-A*36:01; HLA-A*01:01; HLA-A*30:04; HLA-A*80:01; HLA-A*30:02/ HLA-B*15:03** |
|  | K**YMDYLSTQVRYL**GE | YMDYLSTQV/YLSTQVRYL | **HLA-A*02:01; HLA-A*02:02; HLA-A*02:11; HLA-A*02:22; HLA-A*02:34/HLA-A*02:02; HLA-A*02:05; HLA-A*02:22** |
