# Supplementary material for: Comprehensive analysis of early T cell responses to acute Zika Virus infection during the first epidemic in Bahia, Brazil
Source: PLoS One. 2024 May 9;19(5):e0302684. doi: 10.1371/journal.pone.0302684 (PMC11081376; doi:10.1371/journal.pone.0302684)
Supplement: S4 Table — (DOCX) [file pone.0302684.s006.docx]

**Supplemental Table 4: Peptide sequence for Zika virus**

| **Protein** | **Sequence** | **Protein** | **Sequence** |
| --- | --- | --- | --- |
|  | VARVSPFGGLKRLPA |  | TDGVYRVMTRRLLGS |
|  | GLKRLPAGLLLGHGP |  | TRRLLGSTQVGVGVM |
| **Capsid** | HGPIRMVLAILAFLR |  | HTMWHVTKGSALRSG |
|  | AILAFLRFTAIKPSL |  | PYWGDVKQDLVSYCG |
|  | AAMLRIINARKEKKR |  | VIKNGSYVSAITQGR |
|  | SNMAEVRSYCYEASI |  | FEPSMLKKKQLTVLD |
|  | QPENLEYRIMLSVHG |  | EAIKTRLRTVILAPT |
|  | EPRTGLDFSDLYYLT |  | MGEAAAIFMTATPPG |
|  | GLDFSDLYYLTMNNK | **NS3** | AAIFMTATPPGTRDA |
|  | WLVHKEWFHDIPLPW |  | RDAFPDSNSPIMDTE |
| **Envelope** | KEALVEFKDAHAKRQ |  | EVPERAWSSGFDWVT |
|  | VSYSLCTAAFTFTKI |  | HSGKTVWFVPSVRNG |
|  | AETLHGTVTVEVQYA |  | SRKTFETEFQKTKHQ |
|  | NSKMMLELDPPFGDS |  | ISEMGANFKADRVID |
|  | IVIGVGEKKITHHWH |  | AGPMPVTHASAAQRR |
|  | KRMAVLGDTAWDFGS |  | WLEARMLLDNIYLQD |
|  | VLGDTAWDFGSVGGA |  | EQRKTFVELMKRGDL |
|  | HPDSPRRLAAAVKQA |  | GDLPVWLAYQVASAG |
|  | SVSRMENIMWRSVEG |  | AQLPETLETIMLLGL |
|  | RGPQRLPVPVNELPH | **NS4A** | GTVSLGIFFVLMRNK |
|  | FVRAAKTNNSFVVDG |  | IGKMGFGMVTLGASA |
|  | CPLEHRAWNSFLVED |  | IDLRPASAWAIYAAL |
| **NS1** | GFGVFHTSVWLKVRE |  | PASAWAIYAALTTFI |
|  | NDTWRLKRAHLIEMK |  | TFITPAVQHAVTTSY |
|  | EMKTCEWPKSHTLWA |  | VLFGMGKGMPFYAWD |
|  | SDLIIPKSLAGPLSH |  | MPFYAWDFGVPLLMI |
|  | RSTTASGRVIEEWCC | **NS4B** | AIILLVAHYMYLIPG |
|  | ECTMPPLSFRAKDGC |  | YMYLIPGLQAAAARA |
|  | STSMAVLVAMILGGF |  | DIDTMTIDPQVEKKM |
|  | AKLAILMGATFAEMN |  | IAVAVSSAILSRTAW |
|  | FKVRPALLVSFIFRA |  | WGEAGALITAATSTL |
|  | PALLVSFIFRANWTP |  | GALITAATSTLWEGS |
| **NS2A** | GDLMVLINGFALAWL |  | RGSYLAGASLIYIVT |
|  | VVPRTDNITLAILAA |  | NQMSALEFYSYKKSG |
|  | LPFVMALGLTAVRLV |  | LVERGYLQPYGKVID |
|  | SGKRSWPPSEVLTAV |  | WNIVRLKSGVDVFHM |
|  | SWPPSEVLTAVGLIC |  | CDIGESSSSPEVEEA |
|  | LLIVSYVVSGKSVDM |  | CPYTSTMMETLERLQ |
|  | PMREIILKVVLMTIC |  | RLQRRYGGGLVRVPL |
|  | VVLMTICGMNPIAIP |  | RNSTHEMYWVSGAKS |
| **NS2B** | TICGMNPIAIPFAAG |  | IRSEHAETWFFDENH |
|  | MNPIAIPFAAGAWYV |  | HAETWFFDENHPYRT |
|  | AIPFAAGAWYVYVKT |  | YRTWAYHGSYEAPTQ |
|  | WYVYVKTGKRSGALW | **NS5** | GIAMTDTTPYGQQRV |
|  | KRSGALWDVPAPKEV |  | QVMSMVSSWLWKELG |
|  | KGETTDGVYRVMTRR |  | EKEWKTAVEAVNDPR |
|  |  |  | AKGSRAIWYMWLGAR |
|  |  |  | RAIWYMWLGARFLEF |
|  |  |  | GARFLEFEALGFLNE |
|  |  |  | IIKYTYQNKVVKVLR |
|  |  |  | QVVTYALNTFTNLVV |
|  |  |  | EVLEMQDLWLLRRSE |
|  |  |  | TQEWKPSTGWDNWEE |
|  |  |  | AKSYAQMWQLLYFHR |
|  |  |  | KYMDYLSTQVRYLGE |

**Continued from Supplemental Table 4: Peptide sequence for Dengue virus**

| **Protein** | **Sequence** |
| --- | --- |
| **Capsid** | VTLLVLIPTV |
| **Capsid** | AFIAFVRF |
| **NS3** | MIIVDEAHF |
| **NS4B** | NIQTAINQV |
| **POLYPROTEIN** | DPASIAARGY |
| **POLYPROTEIN** | TPEGIIPALF |
| **POLYPROTEIN** | DTTPFGQQR |

**Continued from Supplemental Table 4: Peptide sequence for Yellow-fever virus**

| **Protein Yellow-fever** | **Sequence** |
| --- | --- |
|  | AHCIGITDRDFIEGV |
|  | GITDRDFIEGVHGGT |
|  | PAEARKVCYNAVLTH |
|  | RKVCYNAVLTHVKIN |
|  | YNAVLTHVKINDKCP |
|  | LTHVKINDKCPSTGE |
|  | WREMHHLVEFEPPHA |
|  | HHLVEFEPPHAATIR |
|  | EFEPPHAATIRVLAL |
|  | PKGAPCKIPVIVADD |
| **Env** | PCRIPVIVADDLTAA |
|  | PVIVADDLTAAINKG |
|  | KINDKCPSTGEAHLA |
|  | KCPSTGEAHLAEENE |
|  | WQSGSGGVWREMHHL |
|  | SGGVWREMHHLVEFE |
|  | ADDLTAAINKGILVT |
|  | TAAINKGILVTVNPI |
|  | NKGILVTVNPIASTN |
|  | LVTVNPIASTNDDEV |
|  | NPIASTNDDEVLIEV |
|  | STNDDEVLIEVNPPF |
|  | DEVLIEVNPPFGDSY |
| **NS1** | GEIHAVPFGLVSMMI |
|  | TRVYMDAVFEYTIDC |
|  | PLVALTLTSYLGLTQ |
|  | LTQPFLGLCAFLATR |
| **NS2** | SLALVGAALHPFALL |
|  | VGAALHPFALLLVLA |
|  | LHPFALLLVLAGWLF |
|  | SGREVIDAMCHATLT |
|  | VIDAMCHATLTYRML |
|  | MCHATLTYRMLEPTR |
|  | PSEPWNTGHDWILAD |
| **NS3** | WNTGHDWILADKRPT |
|  | VLVDEGRKVAIKGPL |
|  | LAECARRRLRTLVLA |
|  | ARRRLRTLVLAPTRV |
|  | HGLDVKFHTQAFSAH |
|  | GEAMDTISVFLHSEE |
| **NS4** | IVMLFILAGLLTSGM |
|  | IGCAMLHWSLILPGI |
|  | SLLWNGPMAVSMTGV |
|  | TDTTPFGQQRVFKEK |
| **NS5** | AKGSRAIWYMWLGAR |
|  | GARYLEFEALGFLNE |
|  | CVVRPIDDRFGLALS |
| **Capsid** | LRKVKRVVASLMRGL |

**Continued from Supplemental Table 4: Peptide sequence for Chikungunya virus**

| **Protein** | **sequence** | **Protein** | **sequence** | **Protein** | **sequence** |
| --- | --- | --- | --- | --- | --- |
| **Capsid** | MEFIPTQTFYNRRYQPRPWA | **Envelope** | STKDNFNVYKATRPYLAHCP | **NsP1** | MDPVYVDIDADSAFLKALQR |
|  | YNRRYQPRPWAPRPTIQVIR |  | ATRPYLAHCPDCGEGHSCHS |  | DSAFLKALQRAYPMFEVEPR |
|  | PRPTIQVIRPRPRPQRQAGQ |  | DCGEGHSCHSPIALERIRNE |  | AYPMFEVEPRQVTPNDHANA |
|  | RPRPQRQAGQLAQLISAVNK |  | PIALERIRNEATDGTLKIQV |  | RQVTPNDHANARAFSHLAIK |
|  | LAQLISAVNKLTMRAVPQQK |  | ATDGTLKIQVSLQIGIKTDD |  | RAFSHLAIKLIEQEIDPDST |
|  | LTMRAVPQQKPRRNRKNKKQ |  | SLQIGIKTDDSHDWTKLRYM |  | IEQEIDPDSTILDIGSAPAR |
|  | PRRNRKNKKQRQKKQAPQND |  | SHDWTKLRYMDSHTPADAER |  | ILDIGSAPARRMMSDRKYHC |
|  | RQKKQAPQNDPKQKKQPPQK |  | DSHTPADAERAGLLVRTSAP |  | RMMSDRKYHCVCPMRSAEDP |
|  | PKQKKQPPQKKPAQKKKKPG |  | AGLLVRTSAPCTITGTMGHF |  | VCPMRSAEDPERLANYARKL |
|  | KPAQKKKKPGRRERMCMKIE |  | CTITGTMGHFILARCPKGET |  | ERLANYARKLASAAGKVLDR |
|  | RRERMCMKIENDCIFEVKHE |  | ILARCPKGETLTVGFTDSRK |  | ASAAGKVLDRNISEKIGDLQ |
|  | ENDCIFEVKHEGKVMGYACL |  | LTVGFTDSRKISHTCTHPFH |  | RNISEKIGDLQAVMAVPDAE |
|  | GKVMGYACLVGDKVMKPAHV |  | ISHTCTHPFHHEPPVIGRER |  | AVMAVPDAETPTFCLHTDVS |
|  | GDKVMKPAHVKGTIDNADLA |  | HEPPVIGRERFHSRPQHGKE |  | PTFCLHTDVSCRQRADVAIY |
|  | KGTIDNADLAKLAFKRSSKY |  | FHSRPQHGKELPCSTYVQST |  | CRQRADVAIYQDVYAVHAPT |
|  | KLAFKRSSKYDLECAQIPVH |  | LPCSTYVQSTAATAEEIEVH |  | YQDVYAVHAPTSLYHQAIKG |
|  | DLECAQIPVHMKSDASKFTH |  | AATAEEIEVHMPPDTPDRTL |  | SLYHQAIKGVRVAYWIGFDT |
|  | MKSDASKFTHEKPEGYYNWH |  | MPPDTPDRTLMTQQSGNVKI |  | RVAYWIGFDTTPFMYNAMAG |
|  | EKPEGYYNWHHGAVQYSGGR |  | MTQQSGNVKITVNGQTVRYK |  | TPFMYNAMAGAYPSYSTNWA |
|  | HGAVQYSGGRFTIPTGAGKP |  | TVNGQTVRYKCNCGGSNEGL |  | AYPSYSTNWADEQVLKAKNI |
|  | FTIPTGAGKPGDSGRPIFDN |  | CNCGGSNEGLTTTDKVINNC |  | DEQVLKAKNIGLCSTDLTEG |
|  | GDSGRPIFDNKGRVVAIVLG |  | TTTDKVINNCKIDQCHAAVT |  | GLCSTDLTEGRRGKLSIMRG |
|  | KGRVVAIVLGGANEGARTAL |  | KIDQCHAAVTNHKNWQYNSP |  | RRGKLSIMRGKKMKPCDRVL |
|  | GANEGARTALSVVTWNKDIV |  | TNHKNWQYNSPLVPRNAELG |  | KKMKPCDRVLFSVGSTLYPE |
|  | SVVTWNKDIVTKITPEGAEEW |  | LVPRNAELGDRKGKIHIPFP |  | FSVGSTLYPESRKLLKSWHL |
|  |  |  | RKGKIHIPFPLANVTCRVPK |  | SRKLLKSWHLPSVFHLKGKL |
|  |  |  | LANVTCRVPKARNPTVTYGK |  | PSVFHLKGKLSFTCRCDTVV |
|  |  |  | ARNPTVTYGKNQVTMLLYPD |  | SFTCRCDTVVSCEGYVVKRI |
|  |  |  | KNQVTMLLYPDHPTLLSYRN |  | SCEGYVVKRITISPGLYGKT |
|  |  |  | HPTLLSYRNMGQEPNYHEEW |  | TISPGLYGKTTGYAVTHHAD |
|  |  |  | GQEPNYHEEWVTHKKEVTLT |  | TGYAVTHHADGFLMCKTTDT |
|  |  |  | VTHKKEVTLTVPTEGLEVTW |  | GFLMCKTTDTVDGERVSFSV |
|  |  |  | VPTEGLEVTWGNNEPYKYWP |  | VDGERVSFSVCTYVPATICD |
|  |  |  | GNNEPYKYWPQMSTNGTAHG |  | CTYVPATICDQMTGILATEV |
|  |  |  | MSTNGTAHGHPHEIILYYYE |  | DQMTGILATEVTPEDAQKLL |
|  |  |  | HPHEIILYYYELYPTMTVVI |  | TPEDAQKLLVGLNQRIVVNG |
|  |  |  | ELYPTMTVVIVSVASFVLLS |  | GLNQRIVVNGRTQRNTNTMK |
|  |  |  | VSVASFVLLSMVGTAVGMCV |  | RTQRNTNTMKNYLLPVVAQA |
|  |  |  | MVGTAVGMCVCARRRCITPY |  | KNYLLPVVAQAFSKWAKECR |
|  |  |  | CARRRCITPYELTPGATVPF |  | FSKWAKECRKDMEDEKLLGI |
|  |  |  | ELTPGATVPFLLSLLCCVRT |  | DMEDEKLLGIRERTLTCCCL |
|  |  |  | LLSLLCCVRTTKA |  | RERTLTCCCLWAFKKQKTHT |
|  |  |  |  |  | WAFKKQKTHTVYKRPDTQSI |
|  |  |  |  |  | VYKRPDTQSIQKVPAEFDSF |
|  |  |  |  |  | IQKVPAEFDSFVVPSLWSSG |
|  |  |  |  |  | VVPSLWSSGLSIPLRTRIKW |
|  |  |  |  |  | SIPLRTRIKWLLSKVPKTDL |
|  |  |  |  |  | LLSKVPKTDLIPYSGDAKEA |
|  |  |  |  |  | IPYSGDAKEARDAEKEAEEE |
|  |  |  |  |  | RDAEKEAEEEREAELTREAL |
|  |  |  |  |  | REAELTREALPPLQAAQDDV |
|  |  |  |  |  | PPLQAAQDDVQVEIDVEQLE |
|  |  |  |  |  | VQVEIDVEQLEDRAGA |

**Continued from Supplemental Table 4: Peptide sequence for EBV virus.**

| **Protein** | **Sequence** |
| --- | --- |
| **BMLF1** | GLCTLVAML |
| **BZLF1** | EPLPQGQLTAY |
|  | RAKFKQLL |
| **EBNA1** | HPVGEADYFEY |
|  | RLRAEAQVK |
|  | RYSIFFDY |
|  | RPPIFIRRL |
|  | VPAPAGPIV |
|  | FRLGRAYGL |
| **EBNA3A** | LEKARGSTY |
|  | YPLHEQHGM |
|  | QAKWRLQTL |
|  | AYSSWMYSY |
|  | VFSDGRVAC |
|  | SVRDRLARL |
|  | AVFDRKSDAK |
|  | IVTDFSVIK |
|  | TYSAGIVQI |
|  | VEITPYKPTW |
| **EBNA3B** | GQGGSPTAM |
|  | HRCQAIRKK |
|  | RRARSLSAERY |
|  | AVLLHEESM |
|  | LLDFVRFMGV |
|  | QPRAPIRPI |
|  | EGGVGWRHW |
|  | LRGKWQRRYR |
|  | RRIYDLIEL |
| **EBNA3C** | HHIWQNLL |
|  | KEHVIQNAF |
|  | FRKAQIQGL |
|  | QNGALAINTF |
|  | EENLLDFVRF |
|  | SSCSSCPLSKI |
|  | TYGPVFMCL |
|  | LLWTLVVLL |
|  | PYLFWLAAI |
| **LMP2** | IEDPPFNSL |
|  | RRRWRRLTV |
|  | VMSNTLLSAW |
|  | LTAGFLIFL |
|  | CLGGLLTMV |
