# Supplementary material for: Comprehensive analysis of early T cell responses to acute Zika Virus infection during the first epidemic in Bahia, Brazil
Source: PLoS One. 2024 May 9;19(5):e0302684. doi: 10.1371/journal.pone.0302684 (PMC11081376; doi:10.1371/journal.pone.0302684)
Supplement: S5 Table — (DOCX) [file pone.0302684.s007.docx]

**Supplemental Table 5: Percentages of identity between ZIKV protein and the 4 DENV serotypes**

| **ZIKV** | | **Identity for DENV Serotypes** | | | | **Median identity for the 4 DENV serotypes** |
| --- | --- | --- | --- | --- | --- | --- |
| **Protein** | **Amino Acid Sequence** | **1** | **2** | **3** | **4** |  |
| **Capsid** | VA**RVSPFGGLK**RLPA | 46% | 46% | 40% | 55% | 46% |
|  | G**LKRLPAGLLL**GHGP | 60% | 60% | 62% | 53% | 60% |
|  | HGPI**RMVLAILAFL**R | 87% | 87% | 87% | 80% | 87% |
|  | AI**LAFLRFTAI**KPSL | 73% | 73% | 73% | 60% | 73% |
|  | A**AMLRIINAR**KEKKR | 67% | 58% | 75% | 58% | 63% |
| **Median identity for each DENV serotype** |  | 67% | 60% | 73% | 58% | 63% |
| **Envelope** | SN**MAEVRSYCY**EASI | 60% | 67% | 53% | 85% | 64% |
|  | QPEN**LEYRIMLSV**HG | 87% | 87% | 87% | 87% | 87% |
|  | EPRT**GLDFSDLYY**LT | 80% | 73% | 80% | 80% | 80% |
|  | GLD**FSDLYYLTM**NNK | 73% | 73% | 80% | 67% | 73% |
|  | WLVH**KEWFHDIPL**PW | 93% | 93% | 93% | 93% | 93% |
|  | KEALV**EFKDAHAKR**Q | 80% | 80% | 87% | 73% | 80% |
|  | V**SYSLCTAAFTFTK**I | 60% | 60% | 60% | 67% | 60% |
|  | A**ETLHGTVTV**EVQYA | 80% | 73% | 80% | 80% | 80% |
|  | NSK**MMLELDPPF**GDS | 67% | 80% | 67% | 80% | 74% |
|  | IVIGV**GEKKITHHW**H | 60% | 60% | 73% | 67% | 64% |
|  | KR**MAVLGDTAW**DFGS | 100% | 100% | 100% | 100% | 100% |
|  | VLG**DTAWDFGSV**GGA | 93% | 93% | 93% | 100% | 93% |
| **Median identity for each DENV serotype** |  | 80% | 77% | 80% | 80% | 80% |
| **NS1** | HPD**SPRRLAAAV**KQA | 80% | 87% | 67% | 73% | 77% |
|  | SVSR**MENIMWRSV**EG | 73% | 80% | 73% | 73% | 73% |
|  | RGPQ**RLPVPVNEL**PH | 40% | 40% | 47% | 67% | 44% |
|  | FV**RAAKTNNSF**VVDG | 60% | 53% | 67% | 53% | 57% |
|  | CP**LEHRAWNSF**LVED | 67% | 73% | 67% | 73% | 70% |
|  | GFGV**FHTSVWLKV**RE | 93% | 93% | 93% | 87% | 93% |
|  | NDT**WRLKRAHLI**EMK | 80% | 87% | 87% | 87% | 87% |
|  | EMKT**CEWPKSHTL**WA | 93% | 87% | 87% | 93% | 90% |
|  | SDLI**IPKSLAGPL**SH | 73% | 93% | 93% | 73% | 83% |
|  | RSTT**ASGRVIEEW**CC | 80% | 93% | 87% | 93% | 90% |
|  | E**CTMPPLSFR**AKDGC | 80% | 80% | 73% | 73% | 77% |
| **Median identity for each DENV serotype** |  | 74% | 79% | 76% | 77% | 77% |
| **NS2A** | STS**MAVLVAMIL**GGF | 62% | 50% | 44% | 78% | 56% |
|  | AK**LAILMGATF**AEMN | 60% | 67% | 47% | 47% | 54% |
|  | FKV**RPALLVSFI**FRA | 60% | 47% | 60% | 53% | 57% |
|  | PALL**VSFIFRANW**TP | 40% | 27% | 40% | 40% | 40% |
|  | GDL**MVLINGFAL**AWL | 0% | 47% | 53% | 67% | 50% |
|  | VVP**RTDNITLAI**LAA | 0% | 0% | 40% | 53% | 20% |
|  | **LPFVMALGLTAVR**LV | 0% | 0% | 67% | 0% | 0% |
|  | SGK**RSWPPSEVL**TAV | 0% | 0% | 0% | 0% | 0% |
| **Median identity for each DENV serotype** |  | 28% | 30% | 44% | 42% | **45%** |
| **NS2B** | SWPP**SEVLTAVGL**IC | 73% | 73% | 73% | 73% | 73% |
|  | LL**IVSYVVSGK**SVDM | 73% | 80% | 73% | 80% | 77% |
|  | PM**REIILKVVL**MTIC | 47% | 47% | 53% | 67% | 50% |
|  | VVL**MTICGMNPI**AIP | 67% | 67% | 60% | 80% | 67% |
|  | TIC**GMNPIAIPF**AAG | 53% | 60% | 47% | 60% | 57% |
|  | M**NPIAIPFAA**GAWYV | 53% | 73% | 47% | 60% | 57% |
|  | AIP**FAAGAWYVY**VKT | 47% | 60% | 47% | 67% | 54% |
|  | W**YVYVKTGKR**SGALW | 60% | 60% | 60% | 89% | 60% |
|  | KRSG**ALWDVPAPK**EV | 0% | 0% | 0% | 0% | 0% |
|  | KG**ETTDGVYRV**MTRR | 0% | 0% | 0% | 0% | 0% |
| **Median identity for each DENV serotype** |  | 47% | 52% | 46% | 58% | 57% |
| **NS3** | TD**GVYRVMTRRLL**GS | 79% | 64% | 64% | 73% | 69% |
|  | T**RRLLGSTQV**GVGVM | 73% | 67% | 67% | 67% | 67% |
|  | HTM**WHVTKGSALR**SG | 73% | 73% | 73% | 73% | 73% |
|  | PYWG**DVKQDLVSY**CG | 73% | 80% | 73% | 73% | 73% |
|  | VIKNGS**YVSAITQGR** | 73% | 73% | 60% | 73% | 73% |
|  | FEPS**MLKKKQLTV**LD | 67% | 73% | 67% | 73% | 70% |
|  | EAI**KTRLRTVI**LAPT | 93% | 87% | 93% | 93% | 93% |
|  | MG**EAAAIFMTA**TPPG | 100% | 93% | 100% | 100% | 100% |
|  | AAIF**MTATPPGTR**DA | 93% | 87% | 93% | 80% | 90% |
|  | RDA**FPDSNSPIM**DTE | 67% | 80% | 73% | 67% | 70% |
|  | EVPE**RAWSSGFDW**VT | 100% | 93% | 93% | 100% | 97% |
|  | HSG**KTVWFVPSVR**NG | 87% | 80% | 87% | 87% | 87% |
|  | SR**KTFETEFQK**TKHQ | 93% | 80% | 87% | 80% | 84% |
|  | I**SEMGANFKA**DRVID | 100% | 100% | 100% | 93% | 100% |
|  | AGP**MPVTHASAA**QRR | 93% | 100% | 93% | 93% | 93% |
|  | WLEA**RMLLDNIYL**QD | 73% | 73% | 73% | 80% | 73% |
|  | EQR**KTFVELMKR**GDL | 93% | 93% | 93% | 100% | 93% |
|  | GD**LPVWLAYQV**ASAG | 93% | 93% | 93% | 100% | 93% |
| **Median identity for each DENV serotype** |  | 85% | 83% | 82% | 84% | **85%** |
| **NS4A** | AQLP**ETLETIMLL**GL | 93% | 86% | 100% | 93% | 93% |
|  | IG**KMGFGMVTL**GASA | 67% | 64% | 67% | 73% | 67% |
|  | G**TVSLGIFFV**LMRNK | 40% | 60% | 40% | 60% | 50% |
| **Median identity for each DENV serotype** |  | 67% | 70% | 69% | 75% | 67% |
| **NS4B** | IDL**RPASAWAIY**AAL | 73% | 80% | 73% | 80% | 77% |
|  | PA**SAWAIYAALTTF**I | 73% | 80% | 73% | 73% | 73% |
|  | TF**ITPAVQHAVTTSY** | 60% | 73% | 60% | 60% | 60% |
|  | VLF**GMGKGMPFY**AWD | 53% | 60% | 53% | 67% | 57% |
|  | MPF**YAWDFGVPL**LMI | 53% | 53% | 53% | 60% | 53% |
|  | AI**ILLVAHYMY**LIPG | 80% | 73% | 73% | 73% | 73% |
|  | YM**YLIPGLQAA**AARA | 60% | 60% | 60% | 60% | 60% |
|  | DID**TMTIDPQVEK**KM | 67% | 73% | 73% | 80% | 73% |
|  | IAV**AVSSAILSR**TAW | 53% | 60% | 53% | 47% | 53% |
|  | W**GEAGALITA**ATSTL | 47% | 47% | 47% | 40% | 47% |
|  | GAL**ITAATSTLW**EGS | 60% | 60% | 60% | 53% | 60% |
|  | RGS**YLAGASLIY**IVT | 83% | 92% | 83% | 83% | 83% |
| **Median identity for each DENV serotype** |  | 64% | 68% | 63% | 65% | 60% |
| **NS5** | NQ**MSALEFYSYK**KSG | 80% | 60% | 73% | 60% | 67% |
|  | L**VERGYLQPY**GKVID | 73% | 67% | 67% | 87% | 70% |
|  | WNIV**RLKSGVDVF**HM | 73% | 93% | 87% | 87% | 87% |
|  | CDI**GESSSSPEV**EEA | 80% | 73% | 87% | 87% | 84% |
|  | CPYTS**TMMETLERL**Q | 80% | 67% | 73% | 73% | 73% |
|  | RLQ**RRYGGGLVR**VPL | 87% | 80% | 87% | 87% | 87% |
|  | RNSTH**EMYWVSGAK**S | 73% | 80% | 73% | 87% | 77% |
|  | IR**SEHAETWFF**DENH | 80% | 73% | 67% | 73% | 73% |
|  | HAET**WFFDENHPY**RT | 80% | 80% | 73% | 80% | 80% |
|  | Y**RTWAYHGSY**EAPTQ | 73% | 73% | 73% | 93% | 73% |
|  | G**IAMTDTTPY**GQQRV | 93% | 93% | 93% | 93% | 93% |
|  | QV**MSMVSSWLW**KELG | 60% | 80% | 67% | 67% | 67% |
|  | E**KEWKTAVEA**VNDPR | 67% | 73% | 53% | 73% | 70% |
|  | AK**GSRAIWYMW**LGAR | 100% | 100% | 100% | 100% | 100% |
|  | RAIW**YMWLGARFL**EF | 100% | 100% | 100% | 100% | 100% |
|  | GARF**LEFEALGFL**NE | 100% | 100% | 100% | 100% | 100% |
|  | IIK**YTYQNKVVKVL**R | 80% | 80% | 80% | 87% | 80% |
|  | QVVT**YALNTFTNL**VV | 73% | 73% | 73% | 80% | 73% |
|  | EV**LEMQDLWLL**RRSE | 33% | 20% | 47% | 40% | 37% |
|  | T**QEWKPSTGW**DNWEE | 80% | 80% | 73% | 73% | 77% |
|  | A**KSYAQMWQLLYF**HR | 93% | 87% | 87% | 87% | 87% |
|  | K**YMDYLSTQVRYL**GE | 67% | 58% | 60% | 58% | 59% |
| **Median identity for each DENV serotype** |  | 78% | 77% | 77% | 81% | 77% |
